# Comparison of measurements obtained with TOF-Cuff placed on the arm and the TOF-Scan on the adductor pollicis muscle during general anaesthesia using mivacurium: a prospective observational clinical trial

**DOI:** 10.1038/s41598-024-76086-6

**Published:** 2024-11-08

**Authors:** Paweł Radkowski, Jakub Ruść, Mariusz Kęska

**Affiliations:** 1 Department of Anesthesiology and Intensive Care, Faculty of Medicine, Collegium Medicum University of Warmia and Mazury, Olsztyn, Poland; 2 Department of Anesthesiology and Intensive Care, Regional Specialist Hospital, Olsztyn, Poland; 3 Regional Specialist Hospital in Olsztyn, Żołnierska 18, Olsztyn, 10- 561 Poland; 4 Department of Human Physiology and Patophysiology, Faculty of Medicine, University of Warmia and Mazury, Olsztyn, Poland

**Keywords:** Outcomes research, Clinical trials

## Abstract

Adequate neuromuscular monitoring of patients is essential to verify complete recovery of neuromuscular function before tracheal extubation. This study aimed to assess the correlation between the values acquired from the brachialis muscle using TOF-Cuff and those obtained from the adductor pollicis muscle with TOF-Scan during anaesthesia with mivacurium. Twenty-five patients were enrolled in the observational clinical trial, with the TOF-Cuff positioned on the upper arm and the TOF-Scan on the thumb. Train-of-four (TOF) values were simultaneously recorded by both devices at 30-second intervals before intubation. Subsequently, measurements were taken every 5 min until the removal of the endotracheal tube. Bland-Altman analyses were conducted to compare assessed endpoints obtained by using the TOF-Cuff and the TOF-Scan. The median onset time measured with TOF-Cuff was numerically longer compared to the TOF-Scan score (120 s vs. 90 s, P = 0.42). Spearman rank correlation revealed a significant positive correlation between onset times measured by TOF-Cuff and TOF-Scan (R = 0.73, P = 0.0001, 95% CI 0.446 to 0.875). For the time to recovery assessed with both methods, Spearman correlation coefficient was R = 0.35 and did not reach statistical significance (P = 0.1). Multiplying the time to recovery from the last dose (according to TOF-Scan) by 0.43 to 2.66, provided concordance with the TOF-Cuff result for the entire range of study group. Conclusion. The concordance between the TOF-Scan on the adductor pollicis and the TOF-Cuff on the upper limb was found to be good. However, both devices showed a false-negative result in patients with clinical symptoms of preterm recovery.

**Supplementary Information:**

The online version contains supplementary material available at 10.1038/s41598-024-76086-6.

## Introduction

Neuromuscular monitoring is suggested as an integral part of standard anaesthetic monitoring to objectively assess the effects of non-depolarising neuromuscular blocking agents (NMBA)^1^. NMBAs are administered during anaesthesia or in intensive care units to ease endotracheal intubation, improve ventilation, and decrease postoperative hoarseness^2^, although they may cause postsurgical respiratory complications that can hinder clinical recovery. ^2,3,4,5^

The resultant motor response is assessed by measuring the acceleration of the thumb. The most employed stimulation pattern is the train-of-four (TOF) which compares motor responses from the fourth and first stimulus and expresses the ratio as a percentage, known as the TOF ratio (TOFR) ranging from 100% (no NMBDs effect) to 0% (no muscle reaction to the fourth stimulus).^6,7^ Recovery from the effect of NMBA is defined as a TOFR ≥ 90%, while residual blockade is TOFR < 90%^8^.

Only acceleromyography devices are currently available as standalone quantitative monitors. Acceleromyography utilizes a piezoelectric sensor to measure muscle or finger acceleration. Movement generates voltage in the transducer, analyzed and displayed on the machine. Initially, monitors measured acceleration in one plane, like the TOF-Watch. Advanced versions, like the TOF-Scan, employ three-dimensional sensors affixed to the thumb, big toe, or brow muscle, measuring acceleration across multiple planes^9^. Additionally, it includes adhesive electrodes positioned over the ulnar nerve, tibial nerve, and the root of the facial nerve, respectively. Adductor pollicis muscle response to ulnar nerve stimulation is a standard neuromuscular blockade monitoring method^1^.

A comparable tool, but based on compressomyography, is the TOF-Cuff. Integrated electrodes within a blood pressure cuff monitor muscle activity in the upper arm when stimulating the plexus brachialis. This method quantifies pressure changes in the cuff, providing equivalent stimulation patterns to TOF-Scan but with simpler application. The TOF-Cuff does not require precise lower arm positioning and is less influenced by patient or staff factors^10^. More research is needed to compare TOF-Cuff with traditional neuromuscular monitoring devices like TOF-Watch and TOF-Scan, and to address unanswered questions about observed differences^7^.

Due to specific clinical need in some patients, we conducted a large clinical study that compared neuromuscular monitoring in different locations. Previoulsy, we published results from the different cohort of the same study^11^. In this paper, we compared the TOF values derived from the brachialis muscle using the TOF-Cuff with those obtained from the adductor pollicis with TOF-Scan after mivacurium administration. We hypothesized no statistically significant differences between measurements from these two neuromuscular monitors. This would allow for the interchangeable utilization of these devices depending on the availability of the locations used to attach their components.

## Methods

### Study design

A group of 25 patients was recruited at the Olsztyn Regional Specialized Hospital according to the following criteria: age 18 to 75, BMI 17–35, ASA physical status I-III and a scheduled surgical procedure. Exclusion criteria comprised: pregnancy and breastfeeding, urgent indications for surgery, ASA physical status > III, neuromuscular diseases, polyneuropathy, diabetes, drug addiction, a family history of malignant hyperthermia, as well as allergies to propofol, fentanyl, or mivacurium. The study rigorously followed the principles outlined in the Helsinki Declaration and was carried out after obtaining written informed consent from all participants and with approval from the Bioethics Commission at the Faculty of Medicine of Collegium Medicum of the University of Warmia and Mazury in Olsztyn (No. 10/2021). Before patient enrolment, the trial was registered on ClinicalTrials.gov (the identifier: NCT04845386, registration date: April 15, 2021, principal investigator: Paweł Radkowski). Clinical decision-making was solely based on the outcomes derived from relaxometry measurements using a TOF-Cuff on the upper extremity. The study was completed according to the protocol.

To find a difference of about 30 s with a 60 s standard deviation (alpha = 0.05, power = 0.80; http://biomath.info/power/index.html), this study was designed to include 30 patients. Ultimately, this cohort included fewer patients than planned, but the power found post hoc for the primary endpoint nevertheless exceeded 90%.

## Anaesthetic procedures

Uniform anaesthetic protocols were implemented for all patients. The whole procedure was perfomed in accordance to the current Good Clinical Practice guidelines^12^. Briefly, monitoring included an electrocardiogram, a non-invasive blood pressure cuff positioned on the upper extremity, pulse oximetry, and capnography. The TOF-Scan sensor was positioned on the thumb, and the electrodes were aligned along the ulnar nerve, located on the inner side of the arm near the wrist, with a spacing of 2 to 5 cm between them. After thorough skin preparation, the TOF-Cuff was placed on one arm (the one without the TOF-Scan sensor) by the manufacturer’s instructions, specifically targeting the shoulder muscles. The amplitude of the train-of-four stimulus was set at 40 mA. Anaesthesia was induced with fentanyl, propofol, lidocaine (if epidural analgesia was not administered), dexamethasone, and mivacurium 0.2 mg/kg. Maintenance of anaesthesia was achieved with sevoflurane at a concentration of 1.0 MAC (Minimum Alveolar Concentration).

Indication for intubation was a TOFR of 0 on the TOF-Cuff. Clinicians were allowed to administer supplementary doses of mivacurium to attain complete spontaneous neuromuscular recovery at the end of the surgical procedure. Hypotension was addressed with ephedrine or a fluid bolus, as clinically necessary. Hypertension was managed by increasing the concentration of sevoflurane.

Supplementary doses of fentanyl were administered as required. Ventilation was regulated to maintain end-tidal carbon dioxide values between 35 and 45 mmHg. Ondansetron was administered 30 min before the end of the surgical procedure. Neostigmine at 40 µg/kg and atropine at 8 µg/kg were given at the conclusion of surgery to ensure the full recovery of neuromuscular function in patients who might not have spontaneously reached a TOFR equal to or greater than 0.9.

## Measurements

Following the patient’s loss of consciousness and discontinuation of mivacurium administration, we initiated simultaneous measurements of TOFR on both the adductor pollicis and the arm. Until intubation, measurements were made every 30 s and then every 5 min until extubation. The endotracheal tube was removed when the TOF-Cuff reading on the arm exceeded 0.9, indicating that the patient was ready to resume independent breathing. We also documented the time of intubation and extubation, repeated doses of mivacurium and side effects. The clinician who conducted the intubation assessed its difficulty on a scale ranging from 1 (very easy) to 4 (very difficult). We measured the onset time (time in seconds from the start of mivacurium injection until emergence of TOFR of 0% – primary outcome), the total recovery time of neuromuscular block (time in minutes from start of mivacurium injection until a normalized TOFR of 90% – primary outcome), relaxation time (time in minutes from the start of mivacurium injection to recovery at TOFR = 90% – secondary outcome), and time to repeated dose (time in minutes from TOFR 0% to repeated dose – secondary outcome).

### Statistical analysis

Due to the lack of normal distribution (assessed by the Shapiro-Wilk test), the distribution of continuous variables was presented as the median, interquartile range, and range. Regarding the dual measurement of the same patients using two methods, the Wilcoxon test was used to assess the differences between them. The relationship between continuous variables was assessed using the Spearman correlation coefficient. For the validation of both methods, the Bland-Altman test was used.

To estimate the correction needed for TOF-Scan, the ratio of the time measured by both methods (TOF-Cuff: TOF-Scan) was calculated, and its distribution was presented using descriptive statistics. The disparity between clinical symptoms of neuromuscular return and measurement results was presented as frequency and percentage. The significance of differences in the number of false negative measurements between the two measurement methods was evaluated using the chi-square test. Also, the significance of proportion differences was calculated. Statistical analysis of the test results was performed in MedCalc Version 22.019. The results were considered statistically significant at *p* < 0.05.

## Results

### Study population

Twenty-five patients were included in the analysis (100% women), with the median age of 61 years (ranged 19–72 years) and a BMI of 27.6 kg/m^2^ (ranged 20.1–33.2 kg/m^2^). Five patients (20%) were classified as ASA grade I, nineteen patients (76%) as ASA grade II, and one patient (4%) as ASA grade III. During the study, patients underwent the following procedures: 18 (72%) laparotomy, 5 (20%) breast surgery, and 2 (8%) strumectomy. Most intubations, 20 out of 25 (80%), were rated as very easy, with 2 (8%) considered easy, and 3 (12%) rather difficult; there was no intubation rated as very difficult or not possible to perform. Nine adverse reactions in the form of redness were reported. Lidocaine was administered in 22 cases and epidural anaesthesia in 3 cases. Two (8%) patients did not reach neuromuscular blockade after 600 s of observation.

## Time to onset

**Fig. 1:**
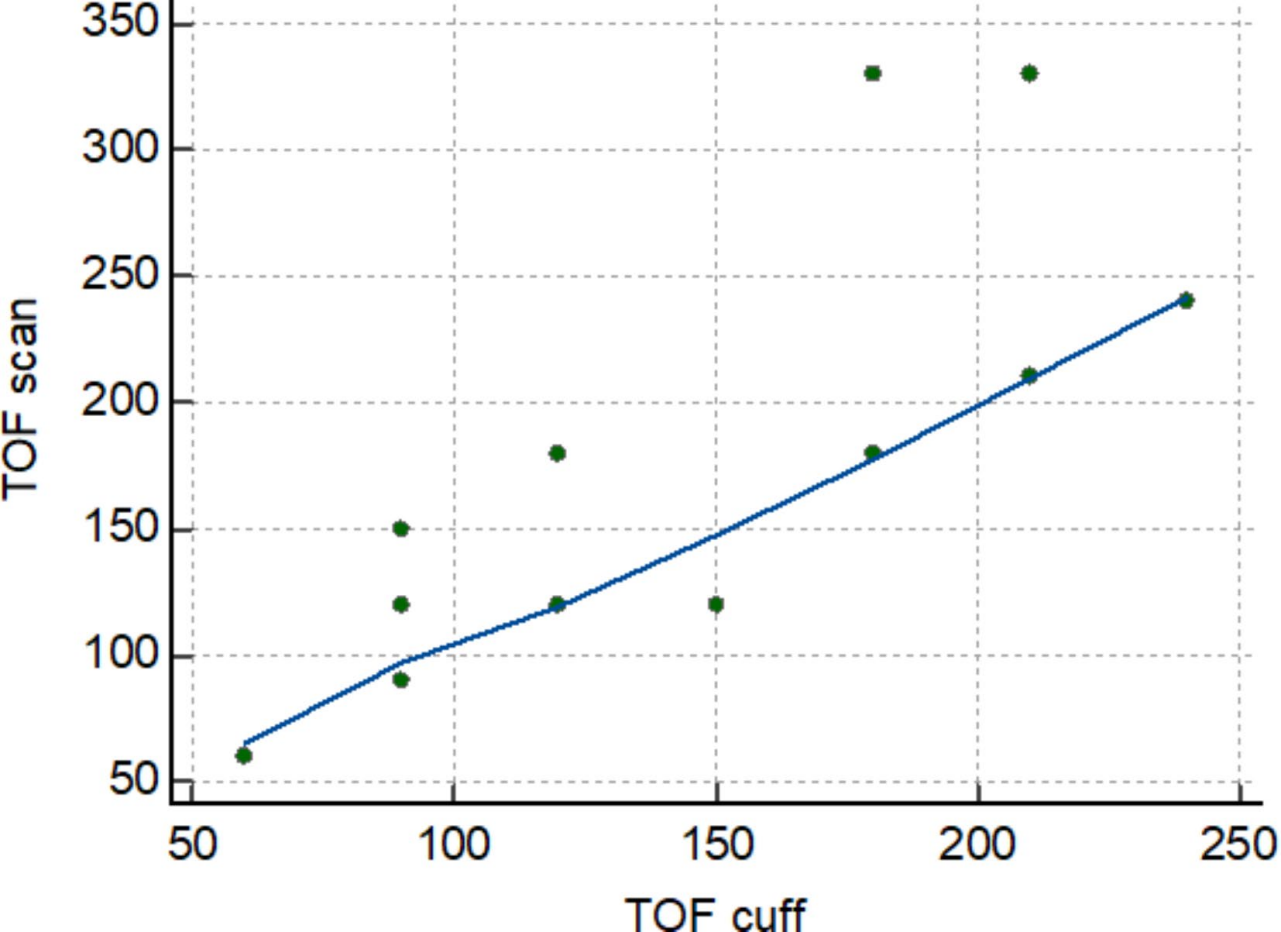
The scatter diagram of Spearman correlation of time to onset between TOF-Scan and TOF-Cuff. The diagram shows data after removing two outlier observations (case 2 and case 19). It shows a strong positive correlation (*R* = 0.9) with statistical significance level *P* < 0.0001. Created in: MedCalc Statistical Software version 22.019.

**Fig. 2:**
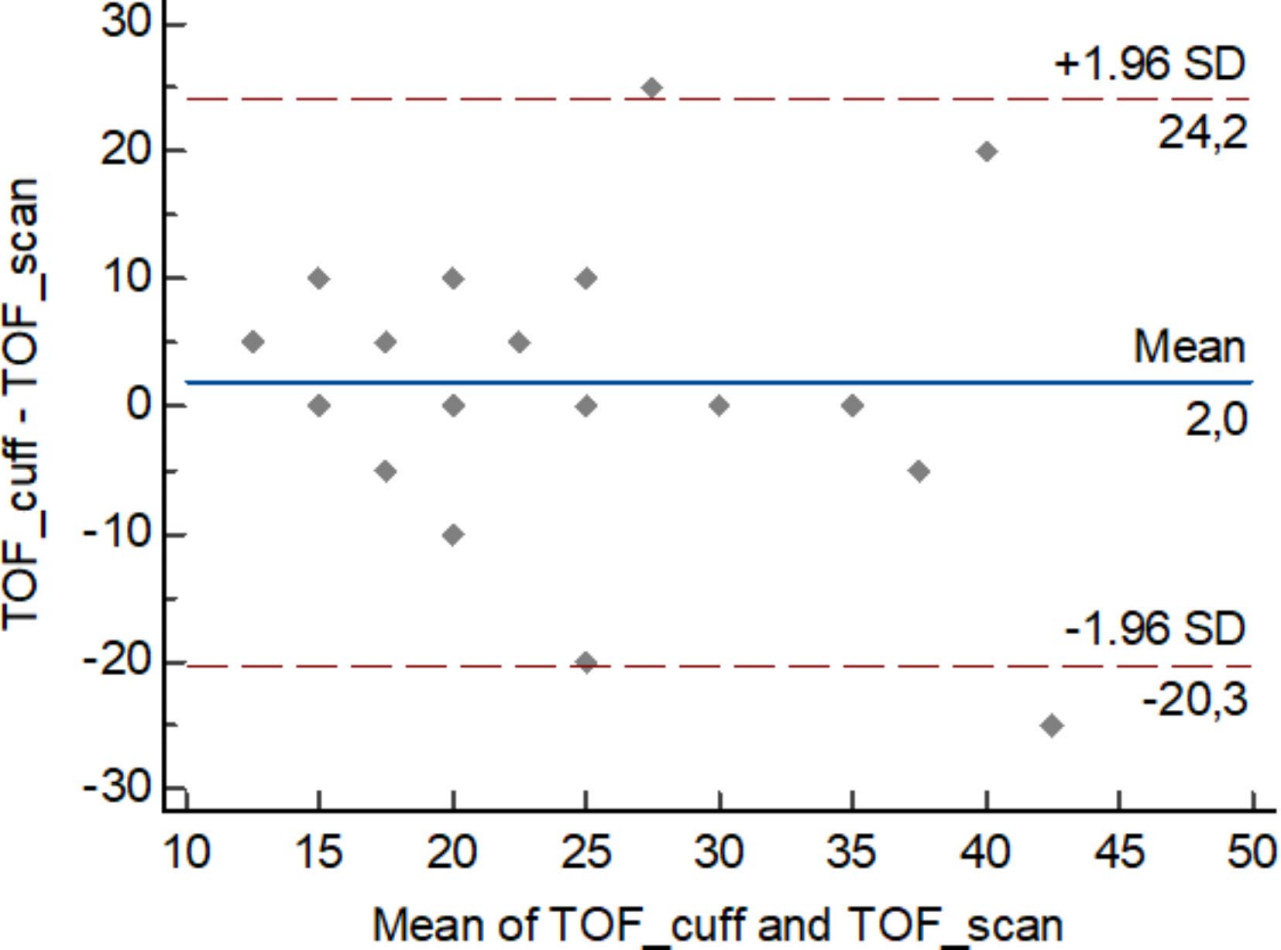
The Bland-Altman plot (average vs. difference) for onset of neuromuscular block. The solid line illustrates the mean difference, and the dashed lines indicate the average difference +/− 1.96 standard deviation of the difference. TOF – train-of-four. (case 2 and case 19). It shows strong positive correlation ( R  = 0.9) with statistical significance level P  < 0.0001. Created in: MedCalc Statistical Software version 22.019.

In the analysis of time to onset, its median value for the brachialis muscle blockage was 120 s, whereas for the hand muscle was 90 s (*p* = 0.42). Median (IQR, range) difference between paired measurements (TOF-Scan – TOF-Cuff) was 30 s (0 s to 75 s, − 180 s to 240 s). Time to onset according to TOF-Cuff as well as TOF-Scan was not dependent on age, BMI, or ASA. The Spearman rank correlation demonstrated a strong positive association between the time to onset in TOF-Cuff and TOF-Scan^®^ measurements (*R* = 0.73, *P* = 0.0001, 95%CI 0.446 to 0.875). The correlation strength increased after excluding two outlier observations (case 2 and case 19) and R reached 0.9 (*P* < 0.0001, 95%CI 0.768 to 0.960). The scatter diagram of the Spearman correlation without two outlier observations is presented in Fig. 1. The Bland-Altman test result was not statistically different (*P* = 0.1). However, after removing two aforementioned outlier observations it revealed a statistically significant consistent bias (*P* = 0.04) (Fig. 2).

Also, we expressed the relationship between the results as the ratio of both measurements. In Table 1, we have presented the time-to-event ratio measured by the two methods, showing what proportion of the time to onset measured with TOF-Cuff is represented by the time to onset measured with TOF-Scan, and therefore by how much the time to onset measured with TOF-Scan needs to be multiplied to obtain a result similar to that measured with TOF-Cuff. The minimum value, successive deciles, and maximum for this correction factor were, respectively: 0.22, 0.55, 0.6, 0.63, 0.67, 0.75, 1.0, 1.25, and 1.57.

**Table 1:** **The time-to-event ratio measured by the two methods (TOF-Cuff and TOF-Scan)**. The number, percentage, and cumulative percentage of patients achieving specified ranges of the quotient of time to TOF_ratio_ = 0 measured by TOF-Scan against the same time measured by TOF-CUFF. TOF – train-of-four. TOF-Cuff – a device designed for neuromuscular monitoring on a brachialis muscle; TOF-Scan – a device designed for neuromuscular monitoring on a hand muscle.

Time to onset rate _TOF−CUFF: TOFF−SCAN_	Number (*N* = 25)	%	Cumulative %
0–1	16	64	64
> 1–2	6	24	88
> 2	1	4	92
Not reached	2	8	100

## Relaxation time

Among the 23 patients, only 6 received a single dose of mivacurium, while 17 (86%) required up to 9 additional doses. In the group of these 6 patients who received only one dose, the relaxation time according to the TOF-Cuff vs. TOF-Scan method was as follows: 10 min vs. 15 min, 10 min vs. 20 min, 10 min vs. 15 min, 15 min vs. 15 min, 8 min vs. 15 min, and 25 min vs. 35 min. The median value was 10 min vs. 15 min, respectively. Due to the small sample size, we refrained from calculating quartiles and we did not evaluate this difference with a statistical test.

When monitoring resumed in the 15th min, TOFR = 0 according to TOF-Cuff was maintained in 13 patients (57%) who had not received an additional dose of mivacurium earlier, while according to TOF-Scan, this was the case for 20 of these patients (87%). In 10 patients (43%), TOFR > 0 was obtained only by TOF-Cuff, and in 3 patients (13%) only by TOF-Scan. A concordant result > 0 according to both methods was achieved in 2 patients (9% of the total patients who did not receive an additional dose in the meantfime), and a concordant result equal to 0 was obtained in 12 patients (52%). Differences in proportions between TOF-Cuff vs. TOF-Scan in the 15th min result were tested. The proportion for a percentage of patients with the TOFR = 0 (57% vs. 87%) and for a percentage of patients with the result > 0 (43% vs. 13%) was both statistically significant (both *P* = 0.025).

In a pooled analysis of patients, regardless of additional doses of mivacurium, median (IQR, range) difference between paired measurements (TOF-Scan – TOF-Cuff) was 1 min (–1 min to 12.5 min, − 12 min to 30 min).

### Time to recovery

**Fig. 3:**
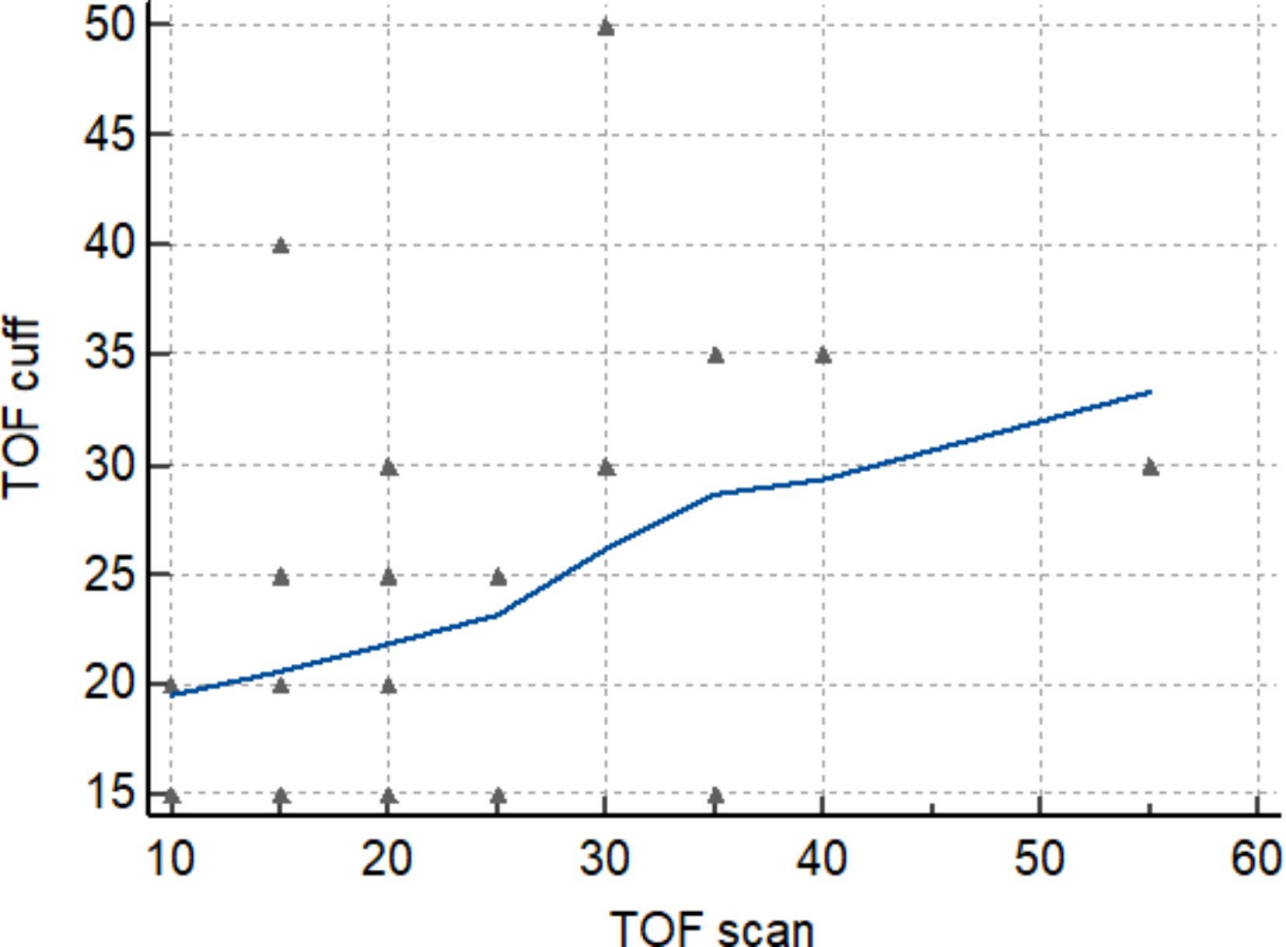
The scatter diagram of Spearman correlation of time from the last dose of mivacurium to TOF_ratio_ >90 between TOF-Scan^®^ and TOF-Cuff^®^. The Spearman’s coefficient of rank correlation *R* = 0.35, and statistical significance was not reached *P* = 0.07. Created in: MedCalc Statistical Software version 22.019.

**Fig. 4:**
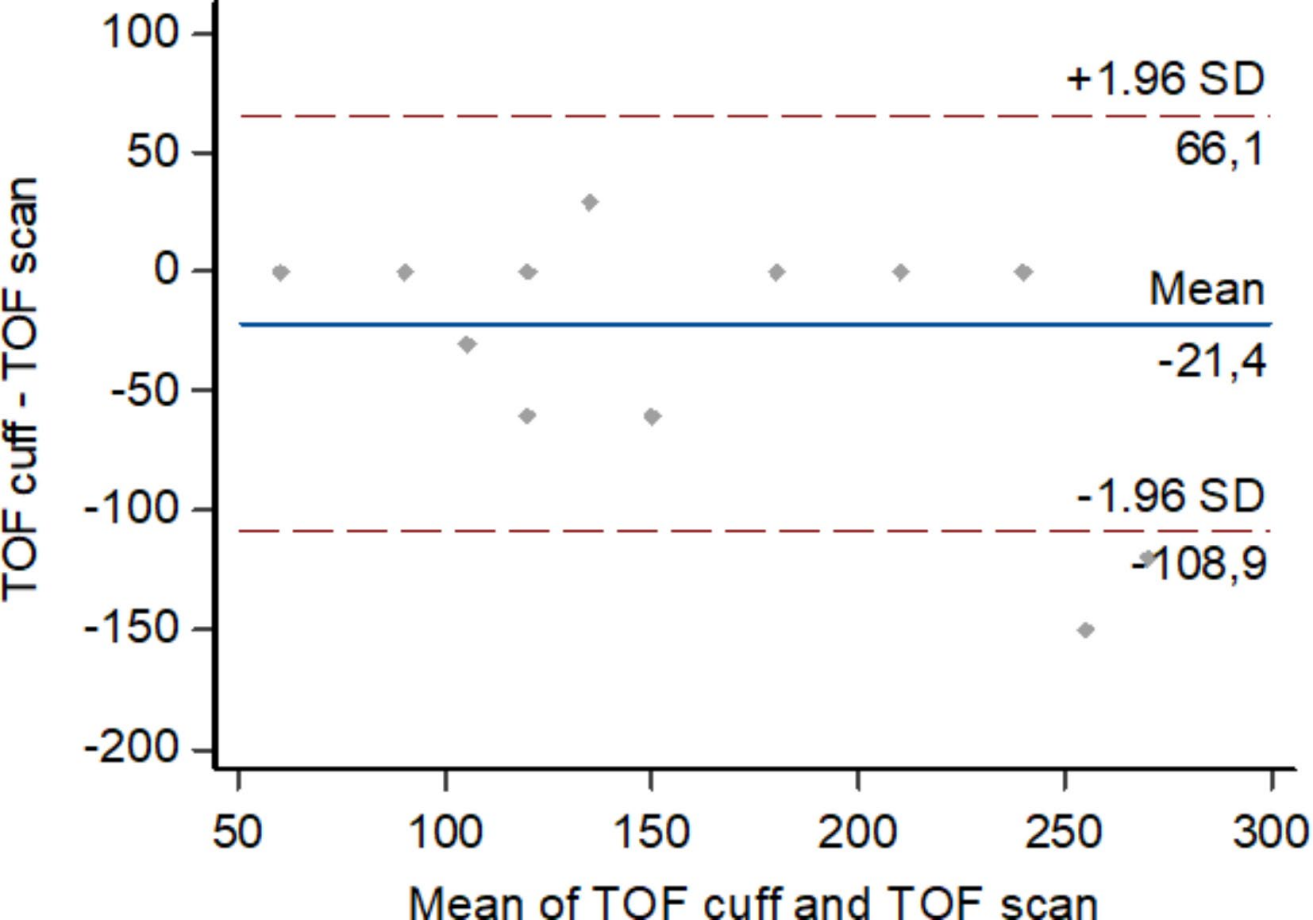
The Bland-Altman plot (average vs. difference) for time from the last dose of mivacurium to TOF_ratio_ >90. The solid line illustrates the mean difference, and the dashed lines indicate average difference +/− 1.96 standard deviation of the difference. Created in: MedCalc Statistical Software version 22.019.

The time from the last dose to TOFR > 0.9 wasn’t significantly shorter for the hand muscle than for the brachialis muscle (median 20 min [10–55 min] vs. 20 min [15–50 min], *P* = 0.3. Median (IQR, range) difference between paired measurements (TOF-Scan – TOF-Cuff) was 5 min (–5 min to 10 min, − 20 min to 30 min). Time to recovery according to TOF-Cuff as well as TOF-Scan was not dependent on age, BMI, or ASA (*P* = 0.67; *P* = 0.45; *P* = 0.55, respectively). Spearman correlation coefficient for time to recovery assessed with both methods was not statistically significant *R* = 0.35 (*P* = 0.1). There was no influence of outlier observations – after excluding two outlier observations (case 12 and case 18) correlation remains weak *R* = 0.04 (95% CI 0.0360 to 0.710) with no significance (*P* = 0.07). The scatter diagram of the Spearman correlation without two outlier observations is presented in Fig. 3. The Bland-Altman plot showed consistent bias (*P* < 0.0001) (Fig. 4).

Similarly to time to onset, we have presented the time to muscular return ratio (Table 2). The minimum value, successive deciles, and maximum for the correction coefficient were, respectively: 0.43, 0.55, 0.57, 0.6, 0.75, 0.88, 1.0, 1.25, 1.33, 1.5, 1.67, 2.0 and 2.66.

**Table 2:** **The time to muscular return ratio.** The number, percentage, and cumulative percentage of patients achieving specified ranges of the quotient of time from the last dose to TOF_ratio_ >90 measured by TOF-Scan against the same time measured by TOF-Cuff. TOF – train-of-four. TOF-Cuff – a device designed for neuromuscular monitoring on a brachialis muscle; TOF-Scan – a device designed for neuromuscular monitoring on a hand muscle.

Time to TOF_ratio_ >90 _TOF−CUFF: TOFF−SCAN_	Number (*N* = 25)	%	Cumulative %
0 to 0.4	0	0	0
> 0.4 to 0.6	5	20	20
> 0.6 to 0.8	2	8	28
> 0.8 to 1.0	6	24	52
> 1.0 to 1.4	4	16	68
> 1.4 to 1.6	2	8	76
> 1.6 to 2.0	5	20	96
> 2.0 to 3.0	1	4	100

### Additional doses of mivacurium

Among 18 patients (44.4%), a total of 76 additional doses of Mivacron were administrated. During these incidents, both methods yielded a false-negative result (TOFR = 0) in 25 cases (32.9%), and both correct result (TOFR > 0) in 18 cases (23.7%); only TOF-Scan^®^ provided an incorrect result in 6 cases (7.9%), and only TOF-Cuff in 27 cases (35.5%). Overall, TOF-Cuff indicated an erroneous result in 45 incidents (59.2%) and a correct result in 31 incidents (48.1%), while TOF-Scan produced an erroneous result in 24 incidents (31.6%) and a correct result in 52 incidents (68.4%) (*P* = 0.0006). In the whole study group, a total of 926 assessments by TOF-Cuff and 939 assessments by TOF-Scan were made from intubation to extubation, indicating a minimal percentage of false-negative indications in both cases (4.9% and 2.6%) of all measurements conducted between intubation and extubation.

## Discussion

This study conducted a comparative analysis of the performance of the TOF-Cuff with the widely utilized clinical accelerometric neuromuscular monitor, TOF-Scan (with a sensor placed on the adductor pollicis muscle) following the administration of neuromuscular blocking agent, mivacurium. We wanted to assess the effectiveness of the TOF-Cuff and, concurrently, verify the importance of arm muscles in monitoring neuromuscular conduction. We observed no statistically significant differences between the onset time, total recovery time, and relaxation time measured by the two devices.

The inclusion of neuromuscular monitoring as standard practice during anaesthesia induction is now supported by numerous anesthesiology societies^13^. This stems from the fact that clinical assessments or baseline nerve stimulators do not accurately represent the patient’s neuromuscular status, and these discrepancies can result in residual neuromuscular blockade before the patient awakens^14^. Nevertheless, despite expert consensus and international recommendations, neuromuscular monitoring is inadequately employed in routine clinical practice^1,15,16^.

The residual neuromuscular blockade is commonly observed after anaesthesia, affecting approximately 40% of patients who received NMBDs during surgery. Although many patients recover from mild muscle weakness without complications, some of them may encounter adverse postoperative events when neuromuscular regeneration is incomplete^5^.

Neuromuscular blockade monitoring can be conducted at various sites by stimulating different nerves and observing the response of the corresponding muscles. So far, the majority of quantitative TOF measurements have been carried out using the ulnar nerve and adductor pollicis. However, if the site is inaccessible or if there’s prior nerve or muscle damage, it can’t be used for monitoring. Since various muscles respond differently to muscle relaxants, it’s clear that optimal values should be established for each anatomical location or muscle group^17^.

Currently, there is limited information or data in the literature regarding the value of the TOF-Cuff monitor. While its application has been described, its advantages, in comparison to standard devices, have not been extensively tested^10^. The device itself appears highly promising as it enables the concurrent utilization of non-invasive blood pressure (NIBP) and neuromuscular conduction monitoring. By placing the electrodes in the NIBP measurement sleeve, it becomes possible to incorporate neuromuscular conduction monitoring at any point during the procedure. This eliminates the need for extra sensor electrodes, leading to cost savings and streamlining the tasks of the anesthesia team^18^.

Some studies undermine the clinical usefulness and safety of the TOF-Cuff for monitoring neuromuscular blockade. Sfeir et al. conducted a trial involving forty patients aged 18–65 years to compare TOF Watch SX with TOF-Cuff and noted that TOF-Cuff significantly overestimated spontaneous recovery times from rocuronium-induced neuromuscular block. Patients monitored with TOF-Cuff alone were shown to be at risk for postoperative residual neuromuscular block^19^.

Kameyama et al. also compared TOF-Cuff and TOF-Watch data after rocuronium-induced neuromuscular block, specifically focusing on elderly patients aged 68–82 years. The study on the other hand indicated that TOF-Cuff may have clinical applicability for assessing both the depth of neuromuscular block and the restoration of neuromuscular function^20^. Kazuma found concordance between the two devices during the onset and pharmacologically enhanced offset of the neuromuscular block but indicated that TOF-Cuff underestimated TOFR in the recovery period, which may have implications for patients’ safety^21^.

In contrast to our results, Markle et al. cautioned against the interchangeable use of TOF-Cuff and TOF-Scan due to significant systematic differences in the time to reach a TOFR of 0%, coupled with substantial intra-individual and clinically relevant variations. In this study, TOF-Cuff demonstrated faster relaxation time and time to recovery compared to TOF-Scan^18^.

A recent study performed by Honing et al. noted a varied discrepancy between the compressomyography at the upper arm and electromyography at the adductor pollicis muscle^4^. The TOF-Cuff was found to overestimate spontaneous neuromuscular block recovery, potentially attributed to the use of a different neuromuscular blocking agent (rocuronium with faster onset time and intermediate duration of action) in a distinct population (involving obese patients who were excluded in our study). Krijtenburg et al. also observed shorter recovery time in TOF-Cuff method^22^.

In our study, we opted to use mivacurium. A dose of 0.20 mg/kg provides favourable intubating conditions within 2–3 min and reaches over 75% TOFR after 29.5–32.1 min^23^. Most studies reported an average onset time for mivacurium ranging from 150 to 240 s^23,24^. Statistical analysis showed that in this cohort of our study, TOFR equal to 0 on the arm occurred on average after 120 s, and on the thumb on average after 90 s. In our previous paper^11^, which described TOF-Cuff vs. TOF-Scan placed on the eyelid, TOFR equal to 0 on the arm occurred on average after 210 s, and on the eyelid on average after 90 s (*P* < 0.00001). In the current study, we obtained the same difference in time to onset. Thus, the time to onset results for TOF-Scan coincide between the thumb and eyelid and are consistently numerically shorter than the time to onset measured with TOF-Cuff. On the other hand, in half of the patients the difference was between 0 and 75 s so its clinical significance was small. For this reason, the correction factor that should be applied to the TOF-Scan results to estimate the TOF-Cuff outcome was significantly smaller in this clinical settings. An above-average variation in some patients, for which no predictor was found among the variables collected, is responsible for the numerical median difference.

A larger numerical difference between the measurements appeared in terms of relaxation time, and this was spodated by the prolonged time to recovery according to TOF-Scan. In the previously described cohort, by contrast, the time to recovery in the TOF-Scan measurement was shortened, suggesting that it is not a different measurement method but a different muscle sensitivity during the recovery phase that is responsible for the difference, which is consistent with previous observations [10, 19, 20]. Symptoms of premature return of muscle function at TOFratio = 0 appeared with both methods, although in this regard, TOF-Scan proved slightly better than the reference method in this clinical trial. This indicates that TOF-Scan is generally a good technological alternative when TOF-Cuff cannot be placed on the arm, but when interpreting the result, it should be taken into account that the biological properties of muscles in different anatomical locations respond differently to the same drug. In addition, despite the lack of statistical significance in the entire study cohort, we noted a large amount of individual variability between patients, which may explain the dissimilarity of our results from the observations of other authors and indicates the need for further research to identify significant predictors to properly qualify patients for an appropriate alternative site when the standard method cannot be used.

Our study has several limitations. First, there is a scarcity of global studies providing information on the use of TOF-Cuff, making it challenging to compare our results. Second, a notable constraint in our study is the researchers’ limited experience with the equipment; any uncertainties or challenges were addressed based on the instructions for use and the manufacturer’s recommendations. Third, our study employed Mivacron, a muscle relaxant with a short duration of action. Presently, an increasing number of countries, including the US and Canada, are opting to phase it out from regular use. Fourth, our patients originated from various departments and underwent diverse procedures, resulting in varying requirements for muscle relaxation and, consequently, distinct doses of muscle relaxants. Fifth, the utilization of short-acting Mivacron for prolonged procedures needed the administration of repeat doses. Additionally, we did not randomize the dominant and non-dominant halves of the body. Nevertheless, this appears to be of minimal importance, due to the lack of significant differences in neuromuscular response between the two arms^25^.

In conclusion, the TOF-Cuff provides an alternative solution to the TOF-Scan, albeit with a consideration for the prolonged onset time, which may elevate the risk of gastric inflation. However, the extended onset time also increases the level of neuromuscular blockade, offering improved intubation conditions. Despite differences in achieving onset time, the time to return to a TOF ratio of 100% is similar between the tested devices suggesting TOF-Cuff can be safely employed for extubating the patient.

**Tables**.

## Electronic supplementary material

Below is the link to the electronic supplementary material.

Supplementary Material 1

Supplementary Material 2

## Data Availability

The data that support the findings of this study are not openly available due to reasons of sensitivity and are available from the corresponding author upon reasonable request.
