# Supplementary material for: Comparison of measurements obtained with TOF-Cuff placed on the arm and the TOF-Scan on the adductor pollicis muscle during general anaesthesia using mivacurium: a prospective observational clinical trial
Source: Sci Rep. 2024 Nov 8;14:27180. doi: 10.1038/s41598-024-76086-6 (PMC11549096; doi:10.1038/s41598-024-76086-6)
Supplement: Supplementary file 1 — Supplementary Material 1 [file 41598_2024_76086_MOESM1_ESM.pdf]

ClinicalTrials.gov PRS DRAFT Receipt (Working Version)
Last Update: 04/09/2021 14:39

ClinicalTrials.gov ID: [Not yet assigned]

Study Identification

Unique Protocol ID: Mivacron 2021

Brief Title: : A Comparison of Neuromuscular Transmission in the Muscles During General Anesthesia.

Official Title: A Comparison of Neuromuscular Transmission in the Arm and Hand Muscles With Alternative Muscles Such as the Lower Leg Muscles and the Corrugator Supercilli During General Anesthesia.

Secondary IDs:

Study Status

Record Verification: April 2021

Overall Status: Enrolling by invitation

Study Start: March 25, 2021 [Actual]

Primary Completion: April 25, 2021 [Anticipated]

Study Completion: June 25, 2021 [Anticipated]

Sponsor/Collaborators

Sponsor: University of Warmia and Mazury in Olsztyn

Responsible Party: Principal Investigator
Investigator: Radkowski Paweł MD PhD [rpaweł]
Official Title: MD PhD
Affiliation: University of Warmia and Mazury in Olsztyn

Collaborators: Wojewódzki Szpital Specjalistyczny in Olsztyn
NOTE : "Wojewódzki Szpital Specjalistyczny in Olsztyn" has been asserted to be a valid organization name by UWMOlsztyn.

Oversight

U.S. FDA-regulated Drug: No

U.S. FDA-regulated Device: No

U.S. FDA IND/IDE: No

Human Subjects Review: Board Status: Approved
Approval Number: 10/2021
Board Name: Komisja Bioetyczna
Board Affiliation: Wydział Lekarski Collegium Medicum Uniwersytetu Warmińsko-Mazurskiego w Olsztynie

Phone: + 48 89 524-53-04
Email: beata.gwiazda@uwm.edu.pl
Address:

Uniwersytet Warmińsko - Mazurski

Katedra Fizjologii i Patofizjologii Człowieka (Szpital Uniwersytecki)

al. Warszawska 30, 10-082 Olsztyn

Data Monitoring: No

FDA Regulated Intervention: No

Study Description

Brief Summary: We want to compare new muscle relaxation monitoring, TOF-Cuff and TOF-Scan, during general anesthesia. Standard monitoring is using thumb, however it can't be use on every patient. Alternative can be eyebrow muscle or foot muscle.

There is only few old study that compares this muscle and used mivacurium. The study aims to compare TOF-Cuff and TOF-Scan with different sensor and use mivacurium.

Detailed Description: In our research in addition to the standard intraoperative monitoring, which includes the assessment of ECG and saturation, we would like to estimate muscle relaxation by using two different methods: a TOF-Cuff which additionally allows to measure inoperative blood pressure and TOF-Scan with different sensor. Our aim is to compare two different methods of muscle relaxation monitoring in patients who will administer muscle relaxant - mivacurium. After receiving painkillers and hypnotics, when the patient is falling asleep, the standard dose of mivacurium 0.2 mg / kg body weight will be administered. Then, painless simultaneous stimulation with TOF-Cuff and TOF-Scan devices will begin. Until intubation, measurements will be made in every 30 seconds and then every 5 minutes until extubation. Extubation will take place according to the guidelines when the TOF-Cuff measurement on the arm is greater than 0.9 - this is the value at which the patient is considered ready to start breathing on his own. The following will be documented: the initiation of drug administration, intubation, possible repeated doses of mivacurium 2 mg, antagonization and possible side effects (e.g., transient cardiac disturbances, reddening of the skin, lowering blood pressure, bronchospasm, erythema, urticaria) and technical problems.

Conditions

Conditions: Neuromuscular Blockade

Keywords: TOF Cuff
TOF Scan
Neuromuscular transmision
corrugator supercilli
Mivacurium
adductor policis

Study Design

Study Type: Observational

Observational Study Model:	Cohort
Time Perspective:	Cross-Sectional
Biospecimen Retention:	None Retained
Biospecimen Description:	
Enrollment:	100 [Anticipated]
Number of Groups/Cohorts:	4

Groups and Interventions

Groups/Cohorts	Interventions
TOF Cuff on arm and TOF Scan on corrugator supercilii<br>Patients undergoing surgery with intubation and receiving a intubation dose of mivacurium (0.2 mg/kg) and repeated dose of mivacurium (2mg) depending of needing under, routine gas anesthesia will have monitoring of neuromuscular block with two monitors simultaneously.	Device: TOFF Cuff on arm<br>The TOF Cuff will be installed on one arm in opposite of TOF Scan. After intubation dose of mivacurium continuous monitoring of neuromuscular block started until complete recovery of neuromuscular block.<br>Device: TOF Scan on corrugator supercilii<br>The TOF Scan will be installed on corrugator supercilii. After intubation dose of mivacurium continuous monitoring of neuromuscular block started until complete recovery of neuromuscular block.
TOF Cuff on lower leg and TOF Scan on adductor pollicis<br>Patients undergoing surgery with intubation and receiving a intubation dose of mivacurium (0.2 mg/kg) and repeated dose of mivacurium (2mg) depending of needing under, routine gas anesthesia will have monitoring of neuromuscular block with two monitors simultaneously.	Device: TOF Cuff on lower leg<br>The TOF Cuff will be installed on one lower leg. After intubation dose of mivacurium continuous monitoring of neuromuscular block started until complete recovery of neuromuscular block.<br>Device: TOF Scan on adductor pollicis<br>The TOF Scan will be installed on Thumb. After intubation dose of mivacurium continuous monitoring of neuromuscular block started until complete recovery of neuromuscular block.
TOF Scan on adductor pollicis and TOF Scan on toe<br>Patients undergoing surgery with intubation and receiving a intubation dose of mivacurium (0.2 mg/kg) and repeated dose of mivacurium (2mg) depending of needing under, routine gas anesthesia will have monitoring of neuromuscular block with two monitors simultaneously.	Device: TOF Scan on toe<br>The TOF Scan will be installed on halux . After intubation dose of mivacurium continuous monitoring of neuromuscular block started until complete recovery of neuromuscular block.<br>Device: TOF Scan on adductor pollicis<br>The TOF Scan will be installed on Thumb. After intubation dose of mivacurium continuous monitoring of neuromuscular block started until complete recovery of neuromuscular block.
TOF Scan on adductor pollicis and TOF Cuff on arm	Device: TOFF Cuff on arm

Groups/Cohorts	Interventions
Patients undergoing surgery with intubation and receiving a intubation dose of mivacurium (0.2 mg/kg) and repeated dose of mivacurium (2mg) depending of needing under, routine gas anesthesia will have monitoring of neuromuscular block with two monitors simultaneously.	The TOF Cuff will be installed on one arm in opposite of TOF Scan. After intubation dose of mivacurium continuous monitoring of neuromuscular block started until complete recovery of neuromuscular block.<br>Device: TOF Scan on adductor pollicis<br>The TOF Scan will be installed on Thumb. After intubation dose of mivacurium continuous monitoring of neuromuscular block started until complete recovery of neuromuscular block.

Outcome Measures

Primary Outcome Measure:

1. Onset time
   Time in seconds from start of injection of mivacurium until emergence of TOF ratio of 0%. TOF = Train of Four

   [Time Frame: 1 to 10 minutes]

2. Total recovery time of neuromuscular block
   The total recovery time, i.e. total duration of the neuromuscular block is defined as the time in minutes from start of injection of mivacurium until a normalized TOF ratio of 90%

   [Time Frame: 30 to 500 min]

Secondary Outcome Measure:

3. Time to repeated dose
   Time in minuted from TOF ratio 0% to repeated dose

   [Time Frame: 5 to 200 min]

Eligibility

Study Population: In the study will take patients qualified to the elective surgery

Sampling Method: Probability Sample

Minimum Age: 18 Years

Maximum Age: 75 Years

Sex: All

Gender Based: No

Accepts Healthy Volunteers: Yes

Criteria: Inclusion Criteria:

Age 18-75 ASA classification I,II,III Elective surgery BMI(body mass index) 17-35 Surgery in the supine position Informed consent of the patient

Exclusion Criteria:

Pregnancy and brestfeeding Urgent indications for surgery ASA classification >III Neuromuscular diseases Polyneuropathy Diabetes Drug addiction Family history of malignant hyoerthermia Allergic to propofol, fentanyl or mivacurium

Contacts/Locations

Central Contact Person: Paweł Radkowski, MD PhD
Telephone: 48 882 815 714
Email: pawelradkowski@yahoo.de

Central Contact Backup:

Study Officials: NOTE : Study Official is required by the WHO and ICMJE.

Locations: **Poland**
Anesthesiology and Intensive Care Clinical Ward, Regional Specialized Hospital
Olsztyn, Warmia-Mazury, Poland, 10-561
Contact: Dariusz Onichimowski, MD PhD +48 89 53 86 243
Principal Investigator: Paweł Radkowski, MD PhD
Principal Investigator: Mariusz Kęska, MD
Sub-Investigator: Jakub Ruść
Sub-Investigator: Agnieszka Barańska, MD

IPDSharing

Plan to Share IPD: Undecided

References

Citations:

Links:

Available IPD/Information:

U.S. National Library of Medicine | U.S. National Institutes of Health | U.S. Department of Health & Human Services
